# A new glaucoma drainage implant with the use of Polytetrafluoroethylene (PTFE). A pilot study

**DOI:** 10.22336/rjo.2021.30

**Published:** 2021

**Authors:** Azadeh Samaeili, Saeed Rahmani, Kiana Hassanpour, Aidin Meshksar, Iman Ansari, Sasha Afsar-Aski, Bahram Einollahi, Mohammad Pakravan

**Affiliations:** * Department of Ophthalmology, Jundishapur University of Medical Sciences, Ahvaz, Iran; ** Department of Optometry, School of Rehabilitation, Shahid Beheshti University of Medical Sciences, Tehran, Iran; *** Ophthalmic Research Center, Research Institute for Ophthalmology and Vision Science, Shahid Beheshti University of Medical Sciences, Tehran, Iran; **** Department of Ophthalmology, Imam Hossein Hospital, Shahid Beheshti University of Medical Sciences, Tehran, Iran; ***** Poostchi Research Center, Shiraz University of Medical Sciences, Shiraz, Iran; ****** Department of Ophthalmology, Labbafinejad Medical Center, Shahid Beheshti University of Medical Sciences, Tehran, Iran; ******* Ophthalmic Epidemiology Research Center, Shahid Beheshti University of Medical Sciences, Tehran, Iran

**Keywords:** Glaucoma filtration surgery, glaucoma drainage implants, polytetrafluoroethylene (PTFE)

## Abstract

**Purpose:** To investigate the implantation of Polytetrafluoroethylene (PTFE) as a glaucoma drainage device.

**Methods:** This study has been done in two steps. First, the constructed implants have been used in 4 rabbits and the histopathologic response was evaluated. In the second step, the implants were used in the 6 eyes of 6 patients with end-stage glaucoma with uncontrolled IOP and poor visual acuity. The tube was made of two-layer of PTFE membrane measuring 8 * 6 mm with a thickness of 1.8 mm and a silicone tube. The rabbits and the human eyes underwent surgical implantation of the tube in the anterior chamber. The histopathologic evaluation was done using H&E staining. Visual acuity, intraocular pressure and the number of glaucoma medications were assessed before and after the surgery.

**Results:** In the histopathologic evaluation, subconjunctival polarizing fibers of a synthetic mesh infiltrated by fibrovascular septa was seen. A granulomatous inflammatory reaction composed of histiocytes, lymphocytes, and multinucleated giant cells were seen around and between the synthetic bundles. The average age of patients was 63 ± 5.5 years. The mean IOP reached from 36.6 ± 5.7 mmHg at baseline to 16.2 ± 8.9 mmHg at the final follow-up. Patients were followed for an average of 6.6 ± 4.5 months. One patient found hypotony refractory to medical and surgical treatment, which led to implant removal. One patient had uncontrolled IOP and finally led to phthisis bulbi following slow CPC. The remaining four eyes did well during the follow-up.

**Conclusion:** The use of PTFE as a new polymer in tube shunt construction was reported. Larger studies, modification of the PTFE membranes like changing the porosity amount, and size of PTFE membranes might result in different conclusions.

## Introduction

Glaucoma drainage implants (GDI) remain an important surgical option in patients with refractory glaucoma. Regarding the clinical outcome, GDI outperforms the trabeculectomy due to a more predictable flow, decreased rate of infection, and lower incidence of hypotony [**1**]. Despite these advantages, all currently available GDIs stimulate a foreign body fibrotic response that leads to a progressive, dense, avascular fibrous capsule formation around the implant [**2**,**3**]. Eventually, the resistance against aqueous outflow increases, the IOP rises, which necessitate anti glaucoma drug administration and the surgery could finally fail [**4**].

Various methods have been used to halt the fibrosis formation, including the creation of a drug-coated device [**5**,**6**], intraoperative or postoperative anti-metabolite [**7**,**8**], subtenon steroid injection [**9**], and early start of aqueous suppressants [**10**]. Furthermore, modification of GDI’s material or design has always been tried [**3**,**11**]. Regarding the design, most GDI consists of a semirigid tube and a rigid and large reservoir. The reservoir plays an essential role in the prevention of the blockage at the distal part. However, the large incision made to insert the reservoir results in a large area of scar formation that increases the failure chance. 

Polytetrafluoroethylene (PTFE), an inert, hydrophobic biocompatible material, has been widely used in cardiovascular and neurosurgery [**12**,**13**]. Ophthalmological application of PTFE includes repair of orbital floor fractures, in frontalis sling procedure [**14**,**15**] and strabismus surgery [**16**]. It was successfully used for vitreoretinal surgery in both rabbit and human eyes and remained stable during the follow-up period without extrusion, migration or inflammatory reaction [**17**,**18**]. PTFE is a porous plate that could hypothetically reduce fibrotic capsule formation while avoiding the complications associated with anti-metabolites use [**19**]. The porous microstructure causes a more diffuse vascular and loose collagen tissue response, instead of the typical avascular, dense collagen reaction [**20**].

In the present study, we aimed to evaluate a PTFE made glaucoma implant in both experimental and human eyes. We hypothesized that PTFE could be a safe material to reduce the fibrous reaction around GDI plates after glaucoma surgery and would adequately control the IOP. 

## Methods

These experimental and clinical interventional case-series were performed in two steps. First, the constructed tube was implanted in the rabbits’ eyes, and in the second step, it was used in the clinical setting in eyes with poor prognosis. The protocol of the study was approved by the ethics committee of the Ophthalmic Research Center, Shahid Beheshti University of Medical Sciences, Tehran, Iran (Registration code: IR.SBMU.ORC.REC.1396.10). The study protocol adhered to the tenets of the Declaration of Helsinki.

*Implant preparation*

**Fig. 1:**
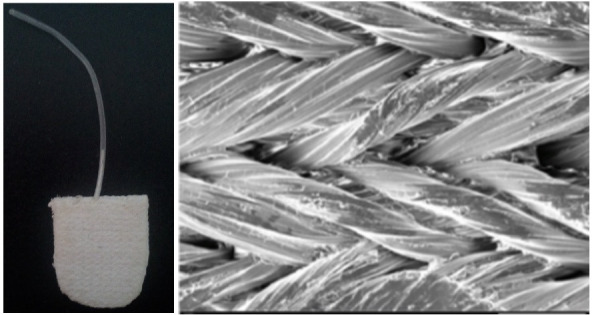
The constructed PTFE implant. Two overlapping plates made of polytetrafluoroethylene (PTFE) measuring 8 * 6 mm with a thickness of 1.8 mm designed for the experimental study forming the reservoir. A silicone tube is inserted between the two layers and fixed with silicone adhesive. The inner diameter of the tube measures 0.3 mm. b) A Scanning Electron-microscopy photograph shows woven fascicles of PTFE consisting of fine microfilaments and disperses coating on PTFE woven fabric so that the whole fiber surface is covered

The implant was made of two overlapping plates of polytetrafluoroethylene (PTFE). Each plate measured 8 * 6 mm with a thickness of 1.8 mm in the rabbit’s eye and 12 * 16 mm in the human eye. After preparing the implant plate, a silicone tube was inserted between the two layers and fixed mechanically. The inner diameter of the tube measured 0.3 mm (**Fig. 1**). The mean pore size of this material has been determined in the previous report to be 167.7 ± 88 microns [**21**]. The implant was sterilized with plasma sterilization before insertion in the rabbits’ eyes.

*Animal preparation*

Four healthy female New Zealand albino rabbits, 10 to 12 months of age, and weighing 2-3 kg, were obtained from Razi Institute for Vaccine and Serum Research, Karaj, Iran. The rabbits were kept under standard conditions (20 ± 1°C, 12 h light/ 12 h dark cycles) and received human care as outlined in the ARVO Statement for the Use of Animals in Ophthalmic and Vision Research.

*Surgical method*

Rabbits were anesthetized with an intramuscular injection of 10% ketamine HCL (Alfamine, 50 mg/ kg, Alfasan, Woerden, Holland) and Xylazine 2% (Rompun, 5 mg/ kg, Bayer, Leverkusen, Germany). After instilling one drop of topical 0.5% tetracaine eye drop (Anestocaine, Sina Darou Laboratories, Tehran, Iran) in the rabbits’ right eyes, a clear cornea traction suture was placed using 7-0 silk. A conjunctival incision was made posterior to the limbus in the superotemporal quadrant. After dissecting conjunctiva and Tenon’s capsule, the PTFE-implant was inserted into the subtenon space and sutured to sclera using two interrupted 7-0 silk sutures 8–10 mm posterior to the limbus.

A 23-gauge needle was used to enter the anterior chamber through a scleral tunnel. The tube was then shortened and inserted into the anterior chamber through the tunnel. A donor scleral patch was placed over the tube and sutured to the sclera with 10-0 nylon. Finally, conjunctiva and Tenon were sutured using a running 8-0 Vicryl. At surgery closure, subtenon antibiotics and steroids were injected in all rabbits’ eyes. The postoperative regimen included topical chloramphenicol 0.5% eye drops (Sina Darou Laboratories Co., Tehran, Iran) four times daily for 1 week and topical betamethasone 0.1% eye drops (Sina Darou Laboratories Co.) six times daily to be tapered gradually over 4 to 6 weeks.

*Clinical investigations*

Clinical observations included wound healing course, scleral and conjunctival redness, and swelling, presence of adhesion and scars, and anterior chamber depth as a possible sign of leakage. These signs at the surgical site were monitored on the 1st, 2nd and 4th post-operative day and continued weekly before enucleation, through gross examination by an expert ophthalmologist. Intraocular pressure was measured before implantation of the GDI and at the time of enucleation by Tonopen (Tono-Pen XL, Reichart Inc., Depew, NY, USA).

*Histopathology*

The animals were euthanized after intracardiac injection of 1 mL Pentobarbital Sodium on the postoperative day 45 and underwent enucleation. After fixation of the eyes in 10% buffered formalin, the whole globe was sent for further pathologic investigation. Sequential 5-µm sections in three different tissue levels (250 µm apart) from the operative wound site (indicated by implant site) were prepared and stained with hematoxylin and eosin (H&E) and examined under light microscopy (BX41, Olympus, Japan) by an ophthalmic pathologist (MRK). Photomicrographs corresponding to the trabeculectomy bleb and subconjunctival implant were captured with a digital camera connected to the light microscope (DP12 Microscope Camera, Olympus, Japan).

*Human Subjects and methods*

Six cases of refractory glaucoma with poor visual potential in one eye, who signed the written informed consent, underwent newly developed glaucoma device implantation. The surgical method was similar to the method described above. The patients underwent a routine ophthalmic examination before and after the PTFE-tube implantation. 

*Statistical analysis*

All statistical analyses were performed using SPSS software (IBM Corp. Released in 2013, IBM SPSS Statistics for Windows, Version 24.0. Armonk, NY: IBM Corp). To describe the data, we used mean and standard deviation (SD). 

## Results

*Histopathological outcomes*

**Fig. 2:**
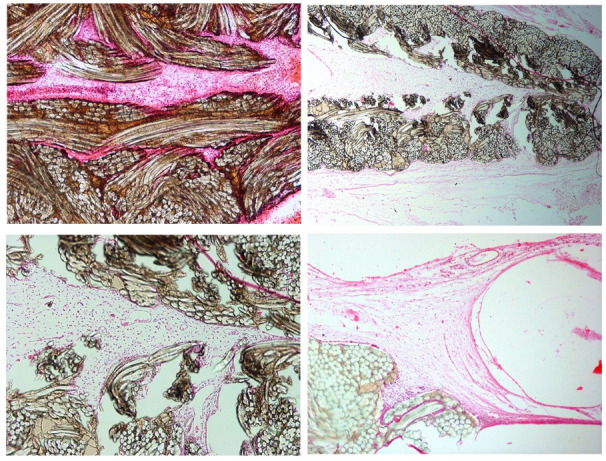
Representative photomicrographs showing subconjunctival polarizing fibers of a synthetic mesh that was infiltrated by fibrovascular septa. Infiltration of a granulomatous inflammatory infiltrate composed of histiocytes, lymphocytes, and multinucleated giant cells are seen around and between synthetic bundles. A cystic bleb-like space surrounded by an epithelial lining is noted at the subconjunctival area over scleral surface adjacent to the sclerocorneal limbus (Staining, H&E)

The whole rabbit globes with implanted PTFE-tube were examined. Microscopic examination revealed subconjunctival polarizing fibers of a synthetic mesh that was infiltrated by fibrovascular septa. Granulomatous inflammatory reaction composed of histiocytes, lymphocytes, and multinucleated giant cells were seen around and between synthetic bundles. A cystic bleb-like space surrounded by an epithelial lining was noted at the subconjunctival area, over the scleral surface, adjacent to the sclerocorneal limbus. A small focal sclerotomy was evident posterior to the corneal limbus. Cornea, iris, retina, and choroid were unremarkable. The result of the histopathologic examination revealed fibro-vascularized subconjunctival mesh together with granulomatous inflammation and a cystic bleb-like space, adjacent to the sclerocorneal limbus (**Fig. 2**).

*Clinical outcomes*

A total of 6 patients with an average age of 63 ± 5.5 years underwent the new shunt implantation. The mean preoperative IOP (median glaucoma medication) was 36.6 ± 5.7 mmHg [**4**] at baseline that reached 16.2 ± 8.9 mmHg [**1**] at the final follow-up (**Table 1**). The eyes had end-stage glaucoma with uncontrolled IOP and poor visual potential, in whom the next surgical plan would have been CPC. The device was removed in one patient and the phthisis bulbi developed in another patient. These two cases were explained in more details. The other four patients did well after 1 year of follow-up. 

**Tabel 1:** Patients’ characteristics and clinical outcomes during the study period

Case	Age	Diagnosis	Pre- IOP	Pre-Meds	Post- IOP	Post- meds	Follow-up time	Final Outcome
#1	61	NVG	30	4	2	0	1 year	Implant removal
#2	55	FHIC NVG	35	4	30	3	1 year	Phthisis Bulbi
#3	67	NVG	36	4	17	0	2 months	No complication
#4	69	NVG	42	3	16	0	2 months	No complication
#5	59	CACG	45	4	14	2	6 months	No complication
#6	67	NVG	32	4	18	2	6 months	No complication
IOP = Intraocular pressure, NVG = Neovascular glaucoma, CACG = Chronic angle-closure glaucoma								

*Case 1*

A 61-year-old man with a history of old ischemic Central Retinal Vein Occlusion (CRVO) of the left eye presented with uncontrolled IOP. He underwent trabeculectomy of the left eye one year before, but the procedure failed despite one episode of needling procedure supplemented by injection of 0.1 cc mitomycin 0.01% (Mitomycin C Kyowa, Kogyo Company, Tokyo, Japan). He had a history of multiple intravitreal bevacizumab injection (IVB). IOP was 30 mmHg on topical dorzolamide hydrochloride 2% and timolol maleate 0.5% (Zilomole, Sina Darou, Tehran, Iran) and brimonidine 0.2% (Brimogan, Sina Darou, Tehran, Iran) three times a day, latanoprost 0.005% (Xalabiost, Bakhtar Bioshimi Pharmaceutical Co. Tehran, Iran) once at night. Visual acuity was 70 centimeters, finger count. So, he was scheduled for PTFE tube implantation and IVB injection. On the 1st postoperative day, IOP was 2 mmHg and the anterior chamber was shallow. He was treated with tropicamide 1% (Mydrax, Sina Daru, Tehran, Iran), Atropine (Atrin, Sina Darou Laboratories, Iran), and phenylephrine 2.5% four times a day and topical chloramphenicol and betamethasone every 6 hours. By the 3rd postoperative day, the anterior chamber became flat; the patient developed vitreous hemorrhage and the visual acuity decreased to light perception, so anterior chamber reformation and tubal ligation were performed for him. The day after visual acuity was hand motion, IOP was 5 mmHg and the anterior chamber was about 2.5 corneal thickness. However, it gradually became shallower and anterior chamber got flat once again by the 7th day. He was scheduled for tube removal and slow Cyclophotocoagulation (CPC). One year later, visual acuity was hand motion and IOP was 12 mmHg, being on Zilomole and brimonidine three times a day and latanoprost once at night.

*Case 2*

The patient was a 55-year-old man known case of Fuchs heterochromic iridocyclitis and NVG of the left eye, who underwent Ahmed glaucoma valve implantation twice, but still had uncontrolled IOP of 34 mmHg, being on Zilomole and Brimogan 0.2% three times a day, latanoprost 0.005% once at night and 250 mg acetazolamide (Iran Daru co., Tehran, Iran) three times a day. Visual acuity was hand motion.

The patient underwent new shunt implantation and intravitreal bevacizumab injection with partial tubal ligation. Due to uncontrolled IOP, slow CPC was performed for him on the 7th postoperative day. On the next day, IOP was 32 mmHg, being on Zilomole and Brimogan three times a day, latanoprost once at night, homatropine 2% (Sina Daru, Tehran, Iran) and betamethasone 0.1% (Sina Daru, Tehran, Iran) every 6 hours and 250 mg acetazolamide three times a day, the visual acuity being hand motion. During the follow-up, he got Epidemic Keratoconjunctivitis (EKC) and was managed with a cold compress and artificial tear. IOP was 12 mmHg on the mentioned topical antiglaucoma medications by the end of the 1st week, after CPC, and remained controlled for 2 months when he developed a sudden onset hypotony (IOP=5 mmHg) and gradually developed phthisis bulbi and visual acuity declined to no light perception (NLP).

## Discussion

The implantation of a new shunt, made of PTFE, has been reported for the first time in the present study. This device resulted in the uncontrolled IOP in 1 eye, refractory hypotony in 1 eye and controlled IOP in the remaining 4 eyes on the short-term. It would be possible that a further continuation of the study led to a different result. However, the poor access to the material made us stop the initial pilot study at this stage.

Modifications to the material, comprising glaucoma surgical devices, have also been explored. Several GDIs are designed to reduce fibrosis formation including a MMC-coated valve [**5**,**6**], or AGV with Adjunctive Amniotic Membrane [**22**]. The use of PTFE is another modification used in new device formation and also in combination with AGV [**19**].

The possible mechanism through which the persistent hypotony occurred could be ascribed to the pore sizes of the implant. Boswell and associates [**23**] histopathologically compared an experimental implant made of ePTFE with conventional Baerveldt implants. They found a significantly thinner capsule around the newly constructed implant and higher vascular profile compared with the Baerveldt implant. They hypothesized that thinner capsules would reduce the failure rate and higher vascular profile would result in higher aqueous absorption. However, the higher vascular profile is another mechanism that could result in the refractory hypotony after the PTFE-tube implantation in our study. Tissue response to a modified AGV implant with an outer ePTFE membrane with a 5-micron pore size was investigated in another experimental study [**19**]. The thinner capsule and highly vascularized tissue were also observed after the application of ePTFE in comparison with the unmodified AGVs. 

Notably, in an experimental study by Bae et al. [**24**], the safety and efficacy of ePTFE made implants have been reported. They continued their trial first in 13 eyes of patients with severe glaucoma [**25**] and then, Kim et al. retrospectively reviewed an ePTFE-made implant in 43 eyes of 40 patients [**26**]. The design of their implant was comparable to ours. Despite their encouraging results, the prevalence of the phthisis bulbi was higher (observed in 3 eyes 7.5%) than in the other reports in their study. The authors attributed the higher rate to the severity of glaucoma in the study population. However, the role of implant design could not be overlooked in this complication. 

In the initial studies reporting the histopathologic investigation of Molteno devices, the rates of phthisis formation were also high [**27**]. However, it has been long since the initial reports of ePTFE declared it as a candidate polymer in the construction of glaucoma shunt, and to date, there has been no prospective trial investigating its efficacy. The ePTFE has been used in the other glaucoma field, including as an implant material for non-penetrating deep sclerectomy (NPDS). Leszczynski et al. [**28**,**29**] investigated the histopathologic outcome of NPDS with e-PTFE implants in two experimental studies. The inhibitory effect of ePTFE on fibroblast activity has been shown in their studies. Although the wound healing process is responsible for fibrosis formation after all filtration surgeries, the mechanism of surgical failure after NPDS is different from tube shunt implantation [**30**]. The fibrosis between Tenon’s capsule and implant forms a membrane that could result in the resistance against the aqueous outflow at the equator of the globe, while the fibrosis formation in the bleb area does not occur near the orbital capillaries responsible for aqueous reabsorption.

A cellular reaction induced by the foreign body and mild fibrovascular response observed in the experimental part of our study is in line with previous histopathological studies reporting the tube implantation [**20**,**23**,**24**]. However, the comparison of capsule thickness and the amount of vascularization around the implant is not possible due to the lack of a control group. Our study is limited by a lack of other staining techniques including Masson’s Trichrome for better demonstration and quantification of fibrosis formation. Another limitation of the present study is a small sample size that places a possible margin of error in our conclusion from PTFE. 

## Conclusion

In conclusion, we reported the use of PTFE as a new polymer in tube shunt construction. This study was designed to serve as a pilot study. Further studies are needed to confirm our results. Modification of the PTFE membranes like changing the porosity amount and the pore size might result in different conclusions.

**Conflict of Interest statement**

The authors declare no conflict of interest.

**Informed Consent and Human and Animal Rights statement**

Informed consent has been obtained from all individuals included in this study.

**Authorization for the use of human subjects**

Ethical approval: The research related to human use complies with all the relevant national regulations, institutional policies, is in accordance with the tenets of the Helsinki Declaration, and has been approved by the institutional review board of Shahid Beheshti University of Medical Sciences, Tehran, Iran.

**Acknowledgements**

We acknowledge our beloved colleague Dr. Mohammad Zare, who passed away from COVID-19 one month prior to this submission. He contributed as an author in this article. His memory is always with us.

**Sources of Funding**

This study was supported by funding received from the ophthalmic research center, research institute for ophthalmology and vision science, Shahid Beheshti University of Medical Sciences, Tehran, Iran.

**Disclosures**

None.
